# Effect of dentin conditioners on biomarker release and microhardness of radicular dentin: an in vitro study

**DOI:** 10.1186/s12903-026-09318-y

**Published:** 2026-09-21

**Authors:** Maii Youssef Elmesellawy, Mona Abdel Rehim Wahby, Ahmed Hussein Abuelezz

**Affiliations:** 1 Beni-Suef University, Beni-Suef, Egypt; 2 Preventive Dentistry, and Dental Public Health Department, Suez University, Suez, Egypt; 3 Misr International University, Cairo, Egypt

**Keywords:** Dentin signaling molecules, Maleic acid, Growth factors, Regenerative endodontics, Microhardness, Conditioning agents

## Abstract

**Aim:**

This study investigated the effects of various radicular dentin conditioning agents, maleic acid, citric acid, phosphoric acid, and EDTA, on dentin-derived biomarker release and radicular dentin microhardness for regenerative endodontic applications.

**Materials and methods:**

Sixty single-rooted teeth were processed into dentin cylinders and discs and randomly assigned to five groups (*n* = 12): 7% maleic acid, 10% citric acid, 37% phosphoric acid, 17% EDTA, and phosphate-buffered saline (PBS) as a negative control. Growth factor release (TGF-β, VEGF, BMP-2) was quantified using ELISA after 24 h incubation. Microhardness changes were evaluated in corresponding dentin cylinders sectioned longitudinally.

**Results:**

Maleic acid produced the highest release of TGF-β, VEGF, and BMP-2 (*p* < 0.001), followed by EDTA, citric acid, and phosphoric acid in a biomarker-dependent hierarchy. Microhardness was significantly affected by the conditioning agent (*p* = 0.004), with EDTA and maleic acid reducing dentin hardness compared with the control, while citric acid and phosphoric acid showed no significant effect. No significant differences were observed among root thirds (*p* = 0.169).

**Conclusion:**

Dentin conditioning in regenerative endodontics involves a trade-off between biological activation and preservation of structural integrity. Stronger conditioning agents enhance growth factor release but may compromise dentin hardness. Among the tested agents, citric acid demonstrated the most balanced performance, combining effective biomarker release with minimal effects on dentin microhardness. In contrast, phosphoric acid preserved dentin structure but showed limited biological activity, making it less suitable when regenerative signaling is the primary objective.

**Clinical relevance:**

The selection of dentin conditioning agents should not be based solely on their biological signaling potential, as it directly influences the mechanical properties of radicular dentin and may impact regenerative outcomes.

## Introduction

Historically, apexification was used for immature, necrotic permanent teeth to induce apical barrier formation to confine filling materials [1]. Regenerative endodontic procedures now aim to preserve tooth vitality, promote continued root development, and restore dentin thickness by replacing necrotic pulp tissue with functional pulp-like tissue [2–5]. Successful regeneration commands a biologically conducive microenvironment that allows stem cell recruitment, scaffold placement, and bioactive signaling [6–9].

Biomarkers, as defined by the National Institutes of Health (NIH), are measurable indicators that reflect underlying biological processes or treatment response [10]. Within the dentin-pulp complex, several bioactive signaling molecules, including growth factors such as transforming growth factor-β (TGF-β) [11, 12], vascular endothelial growth factor (VEGF) [13], and bone morphogenic proteins (BMP) [14–16] are sequestered in the dentin matrix. Once released, these molecules regulate cell homing, proliferation, and differentiation of dental pulp stem cells into odontoblast-like cells, thereby supporting dentinogenesis [17, 18].

Conditioning agents modulate dentin mechanically and chemically by dissolving hydroxyapatite, releasing calcium and phosphate ions, and liberating embedded bioactive signaling molecules [19–22]. Phosphoric acid, a strong acid widely used in restorative dentistry, is investigated here as a novel dentin conditioning agent in regenerative endodontics to enhance biomarker release and modulate dentin structure. Bridging its established clinical role with new insights into dentin-surface interactions and the potential to enhance regenerative outcomes [7, 23–26]. While ethylenediaminetetraacetic acid (EDTA) remains the gold standard for releasing dentin morphogens, it reduces microhardness and alters surface properties, limitations that phosphoric acid, as a novel dentin conditioning agent, may overcome while enhancing biomarker release [20, 21, 24, 27–37]. Citric acid, a weaker organic acid, has been extensively evaluated as a dentin conditioner, offering gentler demineralization and superior biocompatibility, serving as a comparative agent to assess efficacy versus stronger acids. Maleic acid, a mild organic acid, effectively balances efficacy and biocompatibility, representing an alternative conditioner and a benchmark for comparison with phosphoric acid [36, 38, 20, 33, 39].

Given the ongoing controversy regarding the optimal conditioning agent for regenerative procedures, this study investigates phosphoric acid as a novel radicular dentin conditioning agent, alongside maleic acid, citric acid, and EDTA, to evaluate their effects on the release of TGF-β, VEGF, and BMP2, as well as on radicular dentin microhardness. The null hypotheses formulated for this study were that the type of conditioning agent (phosphoric acid, maleic acid, citric acid, or EDTA) would not significantly influence: (1) the release of dentin matrix biomarkers (VEGF, TGF-β, and BMP-2), or (2) dentin surface microhardness.

## Materials and methods

### Sample size determination

The sample size was determined by power analysis with VEGF release as the primary outcome variable. Based on data reported by Khan et al. [37], The mean VEGF values were 161.5, 168.6, and 161.7 pg/mL, respectively, with a within-group standard deviation of 5pg/mL. The calculated effect size (f) was 0.66. Using an α level of 0.05 and a β level of 0.20 (power = 80%), the minimum required sample size was 30 specimens. The calculation was performed using G*Power v3.1.9.2.

### Inclusion and exclusion criteria

All teeth were collected anonymously from clinical extractions performed for periodontal or orthodontic reasons, in accordance with the guidelines of the ethical committee (REC-FDBSU/05012023-01/EM). Sixty extracted human permanent single-rooted teeth with fully developed apices and intact dentin structure were included.

Specimens were required to be free from caries, restorations, cracks, resorption, previous endodontic treatment, or structural defects. Teeth exhibiting anatomical aberrations, canal calcification, fractures, or internal or external resorption were excluded. Residual soft tissue was carefully removed with a scalpel, and teeth were stored in 0.2% sodium azide until use.

### Sample preparation and chemical treatment

All 60 extracted teeth were decoronated to obtain 12 mm-long root cylinders. The root canal lumen of each sample was prepared using a #3 Gates Glidden drill (Dentsply Sirona, Ballaigues, Switzerland) under continuous sterile saline irrigation to standardize the internal lumen diameter across all experimental groups, ensuring a uniform internal geometry for chemical conditioning and subsequent biomarker quantification in this in vitro model [40, 41]. Thirty cylinders were retained intact for microhardness evaluation, with longitudinal grooves created on the mesial and distal surfaces without entering the canal space [42]. The remaining 30 cylinders were sectioned below the cementoenamel junction to produce standardized 2 mm-thick dentin discs for biomarker analysis [37]. Dentin volume and surface area were carefully standardized to minimize variability. Sectioning was performed using a low-speed diamond disk mounted on an Isomet 4000 Precision Cutter (Buehler Ltd., Lake Bluff, IL, USA) under continuous PBS cooling (Sigma-Aldrich, St. Louis, MO, USA) [6, 7, 37].

Each specimen was subjected to a standardized pretreatment sequence of 2 mL 17% EDTA for 3 min, followed by 2 mL of 2.5% sodium hypochlorite. This pretreatment rationale was designed to standardize a clinically realistic, pre-conditioned dentin substrate, ensuring all subsequent active conditioning agents were evaluated under uniform baseline conditions [7, 34, 35, 43–45]. The specimens were then thoroughly rinsed with distilled water.

Subsequently, the dentin discs were stored in 0.5% chloramine solution (BDH Chemicals, UK) and kept for 24 h before use.

### Conditioning of samples

All dentin samples were randomly assigned to five experimental groups based on the conditioning solution; each sample was immersed in 10 mL of the assigned solution for 10 min at room temperature under static conditions. This 10-minute immersion period was selected based on established in vitro regenerative methodologies to allow standardized comparison of growth factor release kinetics and to ensure biomarker concentrations reached quantifiable levels for ELISA detection, acknowledging that it exceeds typical clinical chairside irrigation times [6, 28, 46]. The solutions were not refreshed during the immersion period. Group I received 10 mL of 7% maleic acid (pH 1.2) (Sigma-Aldrich, St. Louis, MO, USA). Group II received 10 mL of 10% citric acid (pH 1.5) (Sigma-Aldrich, St. Louis, MO, USA). Group III received 10 mL of a 37% phosphoric acid aqueous solution (pH ≈ 1), prepared by dilution of an 85% stock solution (Sigma-Aldrich, St. Louis, MO, USA) under controlled laboratory conditions. Group IV received 10 mL of 17% EDTA solution (pH 7.5–8.0) (Sigma-Aldrich, St. Louis, MO, USA), and Group V received 10 mL of phosphate-buffered saline (PBS) (pH 7.4) (Sigma-Aldrich, St. Louis, MO, USA). Following conditioning, specimens were immediately rinsed with 10 mL of distilled water for 30 s to ensure complete removal of residual solution and arrest the conditioning process [28, 34, 35].

### Biomarker preparation and ELISA

After conditioning, each disc was placed in a centrifuge tube containing 1 mL of PBS and incubated at 37 °C for 24 h to allow biomarker release. The eluates were collected in 24-well plates, neutralized to pH 7.0–7.2 using sodium hydroxide, and stored at − 20 °C until analysis [28, 47].

**Fig. 1:**
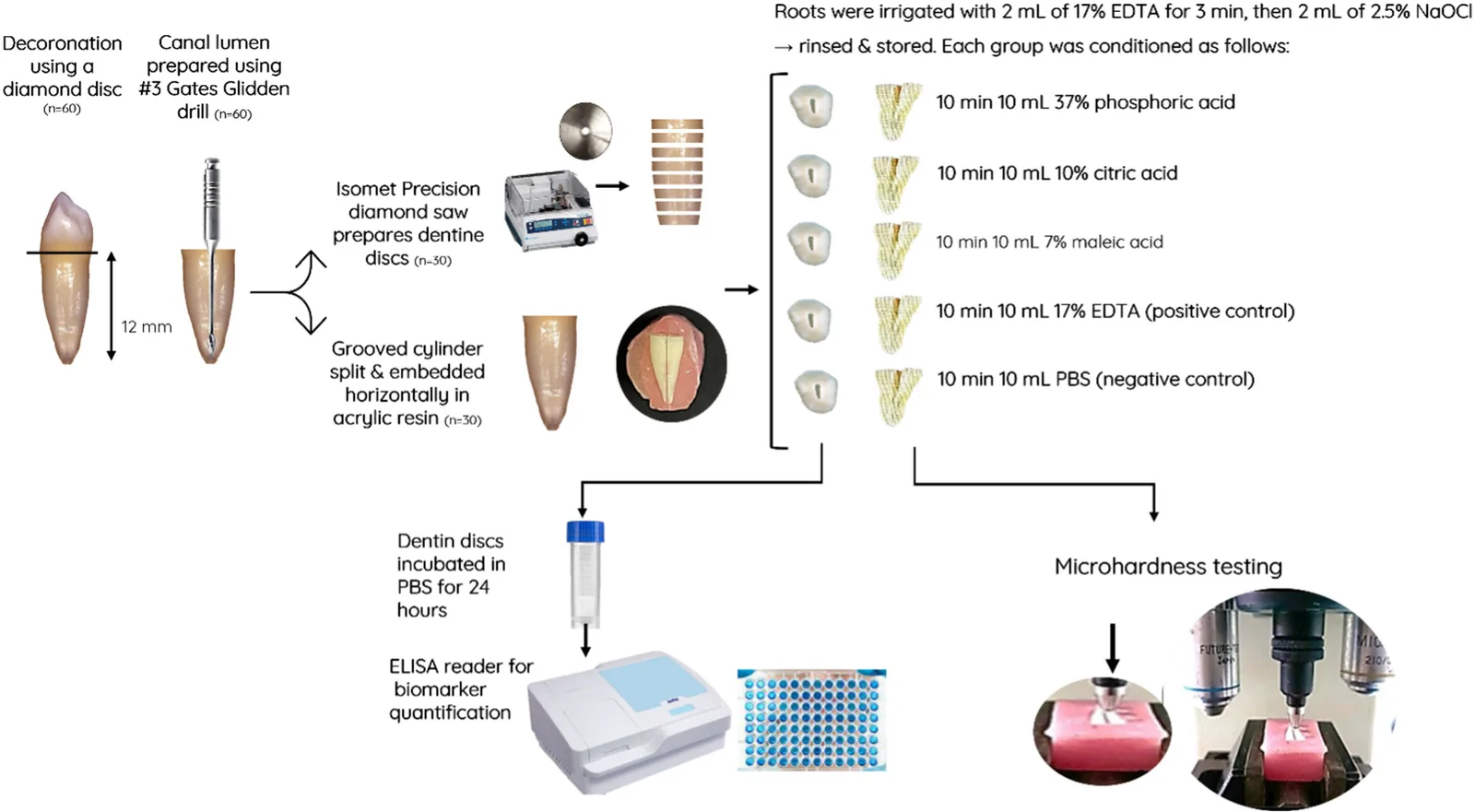
Schematic representation for specimen preparation and testing

Quantification of VEGF, TGF-β, and BMP-2 was performed using enzyme-linked immunosorbent assay (ELISA) kits specific for VEGF and BMP-2 (Elabscience Biotechnology Inc., Houston, TX, USA) and TGF-β (FineTest, Wuhan, China), following the manufacturers’ instructions. Absorbance was measured at 450 nm using a microplate reader (Stat Fax 2100, Awareness Technology Inc., Palm City, FL, USA). All ELISA measurements were duplicated for each sample, and the mean value was used for statistical analysis (Fig. 1).

### Microhardness testing

Thirty root cylinders were used for microhardness evaluation. Longitudinal grooves were created on the mesial and distal surfaces without entering the canal lumen. Specimens were embedded horizontally in self-curing acrylic resin and polished sequentially with silicon carbide abrasive papers (FGM, Brazil) under continuous distilled water to produce smooth, flat surfaces.

Vickers microhardness was measured using a Wilson^®^ Tukon™ 1102 microhardness tester (Buehler Ltd., Lake Bluff, IL, USA). To minimize natural structural variations, three Vickers microhardness indentations were performed in the coronal, middle, and apical thirds, at standardized distances of 500 μm and 1000 μm from the inner canal lumen. A 300 g load (HV 0.3) was applied for 15 s using a square-based diamond pyramid indenter.

The diagonal lengths of each indentation were measured three times, and the mean value was calculated to the nearest 0.1 μm. Vickers hardness number was computed using the following formula: VHN = 1854.4 L/d², where L is the load in Gram-force (gf), and d is the mean diagonal in micrometer (µm), yielding gf/µm² as the hardness value (Fig. 1). For the final analysis, data from these three regional thirds were pooled and averaged to determine a single, representative baseline microhardness value for each experimental group.

### Statistical analysis

Numerical data are presented as mean ± standard deviation (SD). Normality and homogeneity of variance were assessed using the Shapiro–Wilk and Levene tests, respectively, confirming normally distributed populations with homogeneous variances. Microhardness data were analyzed with two-way mixed-model ANOVA, followed by main and simple effects comparisons with p-values adjusted using the False Discovery Rate (FDR) method.

Biomarker expression levels were analyzed with one-way ANOVA and Tukey’s post hoc test. Correlations were evaluated using Pearson’s coefficient. Significance was set at *p* < 0.05. Analyses were performed using R software (v4.3.3, Windows) [48].

## Results

**Fig. 2:**
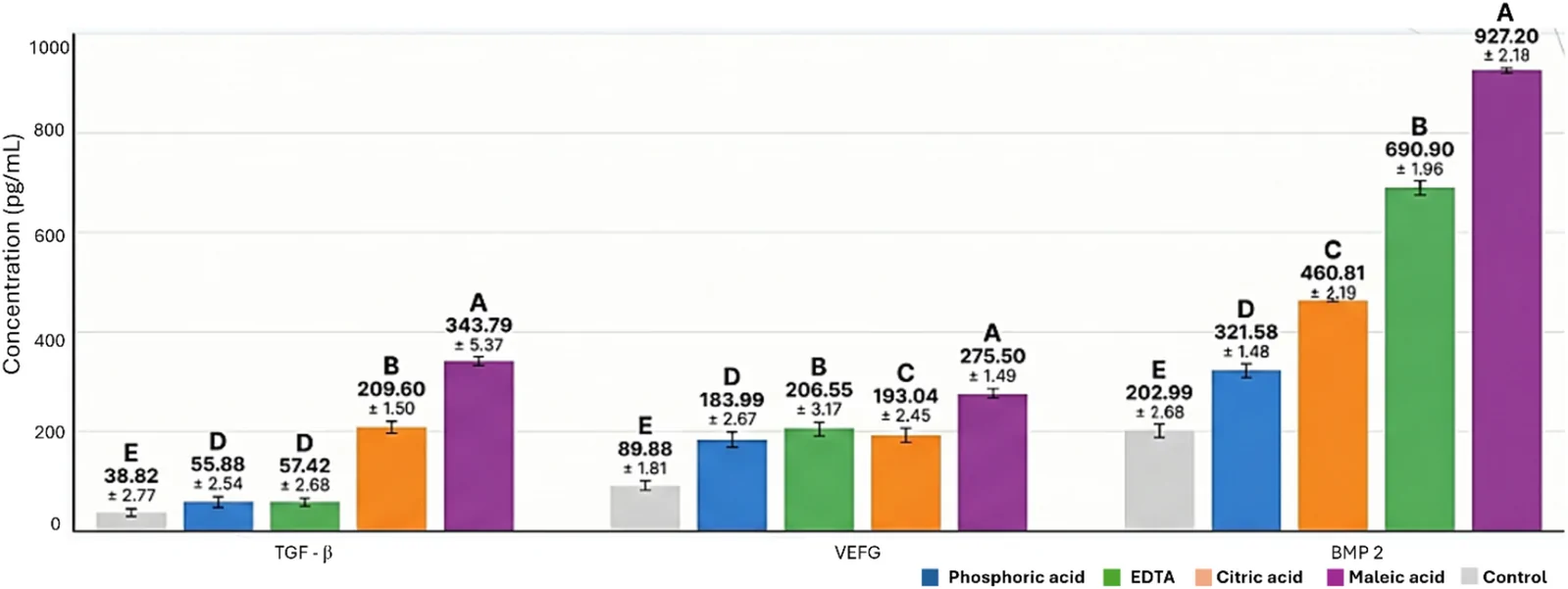
Quantitative release of dentin-derived growth factors, following treatment with various acid conditioners. Data are presented as mean ± SD. For each biomarker, different uppercase letters indicate statistically significant differences between groups (*p* < 0.001): **A** maleic acid; **B** EDTA; **C** citric acid; **D** phosphoric acid; **E** control

The concentrations of VEGF, TGF-β, and BMP-2 released from dentin following surface conditioning are presented in Table 1; Fig. 2. The conditioning agent had a significant effect on the liberation of all biomarkers (*p* < 0.001). Maleic acid consistently produced the highest biomarker yield, eliciting concentrations that were significantly greater than all other groups (*p* < 0.05).

**Table 1:** Summary statistics and intergroup comparisons of the concentrations of different biomarkers (pg/mL)

Biomarker	Phosphoric acid	EDTA	Citric acid	Maleic acid	Control	*p*-value
VEGF	183.99 ± 2.67ᴰ	206.55 ± 3.17ᴮ	193.04 ± 2.45ᶜ	275.50 ± 1.49ᴬ	89.88 ± 1.81ᴱ	< 0.001*
TGF-β	55.88 ± 2.54ᴰ	57.42 ± 2.69ᴰ	209.60 ± 1.50ᴮ	343.79 ± 5.37ᴬ	38.82 ± 2.77ᴱ	< 0.001*
BMP2	321.58 ± 1.48ᴰ	690.90 ± 1.96ᴮ	460.81 ± 4.19ᶜ	927.20 ± 2.15ᴬ	202.99 ± 2.08ᴱ	< 0.001*

Values are presented as mean ± standard deviation (SD)

Different superscript letters within the same row indicate statistically significant differences (*p* < 0.05)

* Indicates a statistically significant overall difference (*p* < 0.05)

For VEGF and BMP-2, EDTA induced a significantly higher release profile than citric acid. Conversely, a distinct, contrasting pattern was observed for TGF-β, where citric acid liberated substantial growth factor concentrations that significantly outperformed EDTA (209.60 ± 1.50 pg/mL vs. 57.42 ± 2.69 pg/mL; *p* < 0.05). Notably, the release of TGF-β following EDTA conditioning remained statistically comparable to that of phosphoric acid.

Phosphoric acid consistently exhibited the lowest biomarker release among the tested agents across all three endpoints, though its values remained significantly higher than those of the untreated negative control (*p* < 0.05).

Dentin surface microhardness values following chemical conditioning are summarized in Table 2. Overall, the conditioning agent had a significant effect on dentin microhardness (*p* = 0.004). No significant interaction or differences were observed between the coronal, middle, and apical root thirds across all groups (*p* = 0.169); therefore, data from the three levels were pooled and expressed as a single mean per group.

**Table 2:** Summary statistics and intergroup comparisons of dentin microhardness (VHN)

Dentin conditioner	Phosphoric acid	Maleic acid	Citric acid	EDTA	Control	*p*- value
Microhardness (Mean ± SD)	45.09 ± 4.22ᴬᴮ	43.42 ± 3.00ᴮ	45.40 ± 4.57ᴬᴮ	42.93 ± 3.57ᴮ	48.52 ± 4.83ᴬ	0.004*

Values are expressed as mean ± standard deviation (SD)

Different superscript letters within the same row indicate statistically significant differences (*p* < 0.05). Data represents the pooled and averaged microhardness values across the coronal, middle, and apical thirds, as regional location did not statistically influence microhardness outcomes (*p* = 0.169)

* Indicates a statistically significant overall difference (*p* < 0.05)

Compared with the untreated control (48.52 ± 4.83), conditioning with 17% EDTA (42.93 ± 3.57) and maleic acid (43.42 ± 3.00) resulted in a significant reduction in microhardness. In contrast, 37% phosphoric acid (45.09 ± 4.22) and 10% citric acid (45.40 ± 4.57) showed no significant difference from the control. However, no statistically significant differences were observed among the four conditioning agents in pairwise comparisons. 

## Discussion

The radicular dentin matrix functions as a bioactive reservoir for signaling molecules that regulate stem cell differentiation, angiogenesis, and mineralized tissue formation [49]. During regenerative endodontic procedures, conditioning agents do not merely remove the smear layer; they actively govern the bioavailability of these matrix-bound growth factors. This biological yield, however, must be critically weighed against potential structural degradation of the root canal wall [43, 50, 51].

Angiogenesis is fundamental to regenerative endodontics, as cellular survival and differentiation within the root canal system depend on an adequate nutrient and oxygen supply. Vascular endothelial growth factor (VEGF) promotes endothelial proliferation and neovascularization, while also supporting dentinogenic activity [52, 53].

Transforming growth factor beta (TGF-β), abundantly released from conditioned dentin, regulates odontoblastic proliferation, differentiation, cell homing, and reparative dentin formation [6, 7, 54, 55]. Bone morphogenetic proteins (BMPs) and TGF-β signaling further regulate odontogenic differentiation of stem cells from the apical papilla and promote matrix formation [56–59].

Within this biological framework, dentin response to conditioning agents reflects chemically driven modulation of growth factor availability, governed by demineralization depth, collagen exposure, and calcium-chelation mechanisms. These interactions likely explain the observed differences in biomarker release profiles, reflecting variable access to matrix-bound proteins without complete structural compromise [28, 60].

Dentin conditioners such as EDTA and organic acids act primarily through controlled calcium removal and demineralization, increasing matrix permeability and exposure of embedded growth factors [40]. In contrast, strongly acidic agents may induce more aggressive demineralization, potentially affecting protein stability and biological detectability [61].

These biochemical effects are closely linked to dentin’s mechanical behavior, as mineral content determines resistance to deformation. Thus, conditioning strategies that enhance biological signaling must be considered in parallel with their effects on structural integrity [62].

The standardized model used in this study employed a uniform EDTA/NaOCl pretreatment across all specimens to simulate a clinically prepared root canal environment. According to the American Association of Endodontists (AAE) principles, such pretreatment removes the smear layer generated during instrumentation, exposing the underlying dentin surface. This ensured a standardized, clinically relevant pre-conditioned substrate for evaluating all conditioning agents under uniform baseline conditions [35, 63].

Among the tested agents, maleic acid demonstrated superior bioavailability of TGF-β, VEGF, and BMP-2, likely reflecting its low pH and high demineralizing capacity, promoting deeper decalcification of peritubular and intertubular dentin, and rapid liberation of matrix-bound morphogens [64]. This substantial biological yield aligns with previous reports describing its superior smear layer removal and effective protein exposure while maintaining favorable cytocompatibility relative to EDTA [33, 65, 66].

Distinct biomarker-specific patterns were also observed. Citric acid triggered substantial liberation of TGF-β, significantly surpassing both EDTA and phosphoric acid, agreeing with previous reports [46, 47]. This specific affinity is consistent with its capacity for controlled intertubular demineralization, facilitating selective exposure of dentin matrix components without denaturing its structure [7, 24, 38, 46, 47, 54, 60, 61]. Conversely, EDTA achieved greater liberation of VEGF and BMP-2, likely due to its pronounced calcium-chelation capacity and ability to unbind more tightly associated matrix proteins. In contrast, phosphoric acid exhibited minimal biological activity for VEGF and BMP-2, which may be related to its aggressive acidic nature and potential protein denaturation under ultra-low pH conditions [28, 36].

These biological behaviors mirror changes in dentin substrate. Dentin hardness, influenced by its mineral content, reflects the tissue’s resistance to deformation and can be altered by exposure to aggressive conditioning agents [19, 43, 68, 69]. Maleic acid and EDTA, which are associated with higher biomarker release, also resulted in significant reductions in microhardness, reflecting increased mineral loss [20, 31, 38, 70, 71]. In contrast, citric acid and phosphoric acid caused only minor, non-significant decreases, indicating superior preservation of structural integrity [19, 72, 73]. These findings are consistent with previous literature, although discrepancies regarding phosphoric acid effects may be attributed to differences in exposure time, concentration, or substrate characteristics [74].

Clinically, these results highlight a clear trade-off between biological activation and structural preservation, supporting rejection of both null hypotheses. Maleic acid maximizes growth factor release but compromises mechanical integrity. Citric acid demonstrates the most balanced profile, achieving meaningful biomarker liberation while preserving dentin structure. Phosphoric acid maintains dentin hardness but provides limited biological stimulation, restricting its suitability for regenerative applications where cellular recruitment is essential.

### Limitations and future directions

Within the limitations of this in vitro study, clinical translation is constrained by the evaluation of a limited biomarker panel and the absence of functional cellular assays. Individual pre-treatment microhardness baseline values were not recorded; however, random allocation of specimens, standardized indentation protocols, and inclusion of an untreated control group were used to reduce inter-sample variability.

In addition, the 10-minute conditioning period exceeds typical clinical irrigation times; while necessary to ensure sufficient demineralization for quantifiable ELISA detection, it may overestimate changes observed under clinical conditions. Furthermore, the standardized EDTA/NaOCl pretreatment ensured baseline uniformity but may have altered the native dentin matrix.

Future studies should incorporate clinically relevant exposure times, baseline mechanical measurements, functional cellular assays, and in vivo validation to improve translational relevance.

## Conclusion

Dentin conditioning in regenerative endodontics involves a trade-off between biological activation and structural preservation. While stronger conditioning agents enhance growth factor release, they may compromise dentin hardness. Citric acid demonstrated the most balanced profile, combining effective biomarker release with preservation of dentin integrity. In contrast, phosphoric acid preserved microhardness but exhibited limited biological stimulation, making it less suitable when biological signaling is prioritized for regenerative purposes.

## Data Availability

The datasets generated and/or analyzed during the current study are available from the corresponding author upon request.
